# Supplementary material for: Hepatocellular Metabolic Abnormalities Induced by Long-Term Exposure to Novel Brominated Flame Retardant, Hexabromobenzene
Source: Toxics. 2023 Jan 21;11(2):101. doi: 10.3390/toxics11020101 (PMC9962401; doi:10.3390/toxics11020101)
Supplement: Supplementary file 1 [file toxics-11-00101-s001.zip › toxics-2133692-supplementary.pdf]

Supplementary information for

Hepatocellular metabolic abnormalities induced by long-term exposure to novel brominated flame retardant, hexabromobenzene

Bohyun Shin, Se Hee Hong, Sumin Seo, Cho Hee Jeong, Jiyu Kim, Eunbin Bae, Donghee Lee, Jung Hoon Shin, Minki Shim, Sang Beom Han*, Dong-Kyu Lee*

College of Pharmacy, Chung-Ang University, 84 Heukseok-ro, Dongjak-gu, Seoul 06974, Republic of Korea

* Corresponding Author:

Sang Beom Han, Ph.D.
College of Pharmacy, Chung-Ang University
84 Heukseok-ro, Dongjak-Gu
Seoul 06974, Republic of Korea
Tel: +92-2-820-5596 Fax: +82-2-3280-5597 E-mail:hansb@cau.ac.kr

* Corresponding Author:

Dong-Kyu Lee, Ph.D.
College of Pharmacy, Chung-Ang University
84 Heukseok-ro, Dongjak-Gu
Seoul 06974, Republic of Korea
Tel: +92-2-820-5854 Fax: +82-2-3280-5854 E-mail:leedk@cau.ac.kr

Figure S1. Morphology of HepG2 cells at a magnification 100×. (A) control; (B) HBB treated for 21 days.

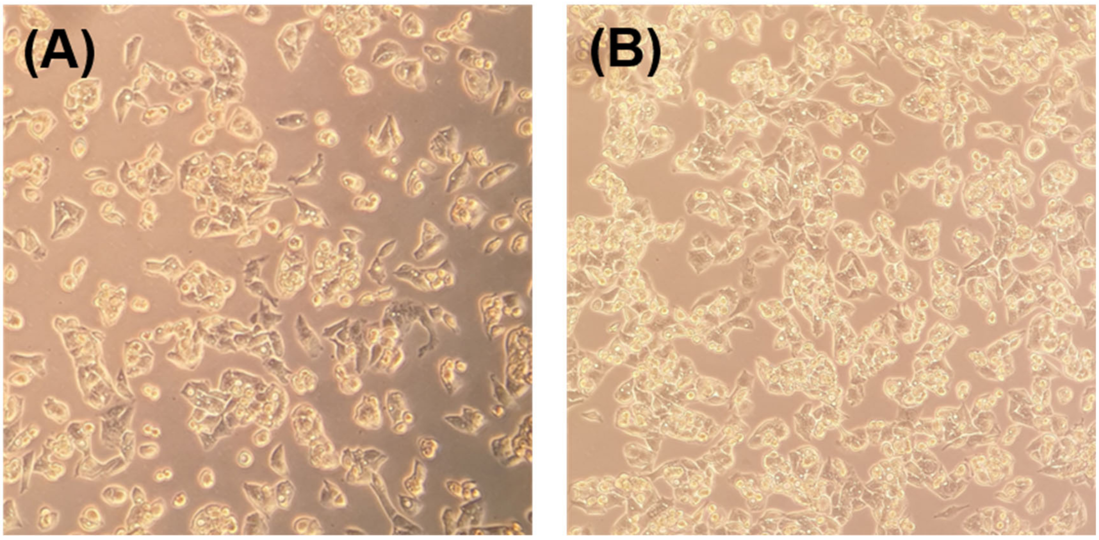

Figure S2. Effect of HBB on HepG2 cell viability. HepG2 cells were exposed to DMSO or HBB (0.05, 0.1, 0.5, 1 μg/mL) for 24 h and 48 h on a culture medium. Data are shown as the mean of the percentage of control ± standard deviation for n = 9, obtained from three independent experiments. (**$p < 0.01$, ****$p < 0.0001$)

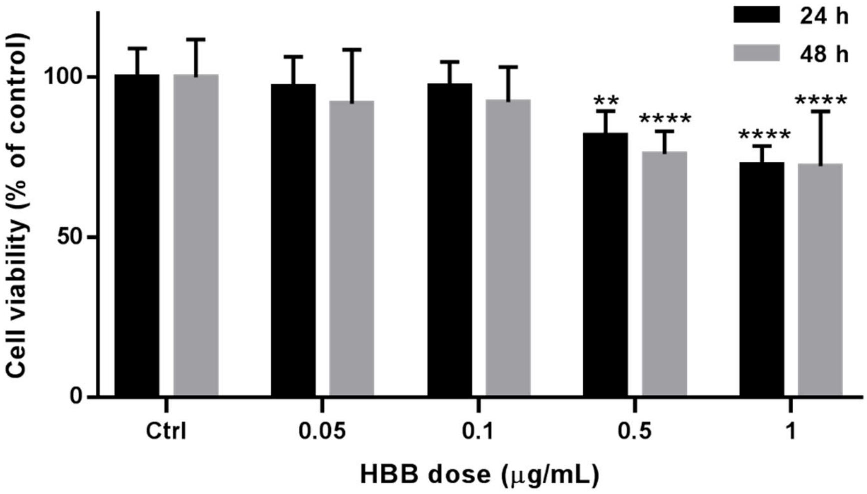

Figure S3. (A) PCA score plots of control group (green, n = 18), HBB-exposed group (blue, n = 18), and QC (red, n = 5) and (B) cross-validation result of PCA.

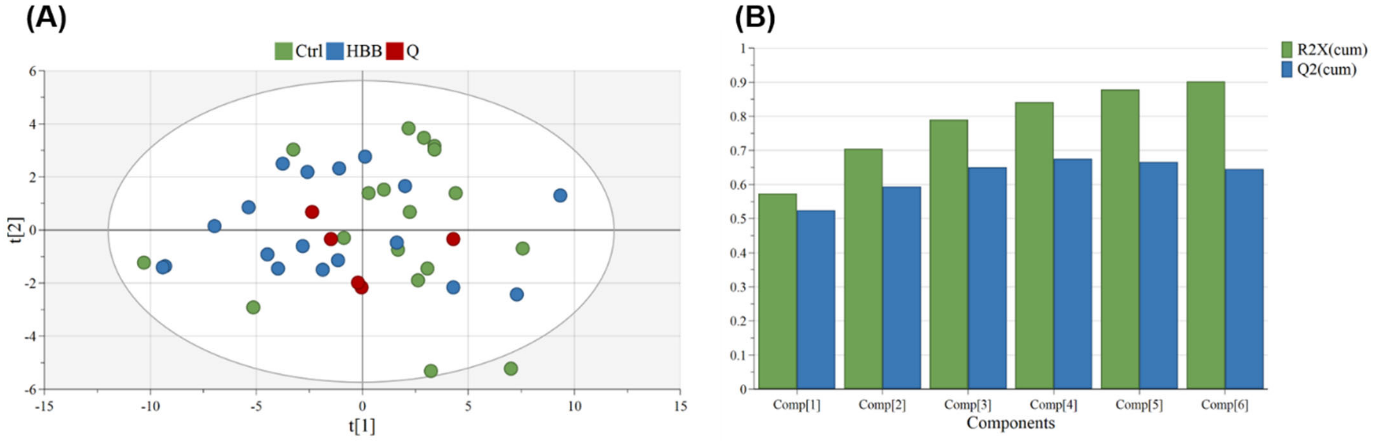

Figure S4. (A) OPLS-DA score plot of short-term (green; 3, 7 d; n = 6) and long-term (blue; 15, 17, 21 d; n = 9) HBB exposure, (B) OPLS-DA permutation test result.

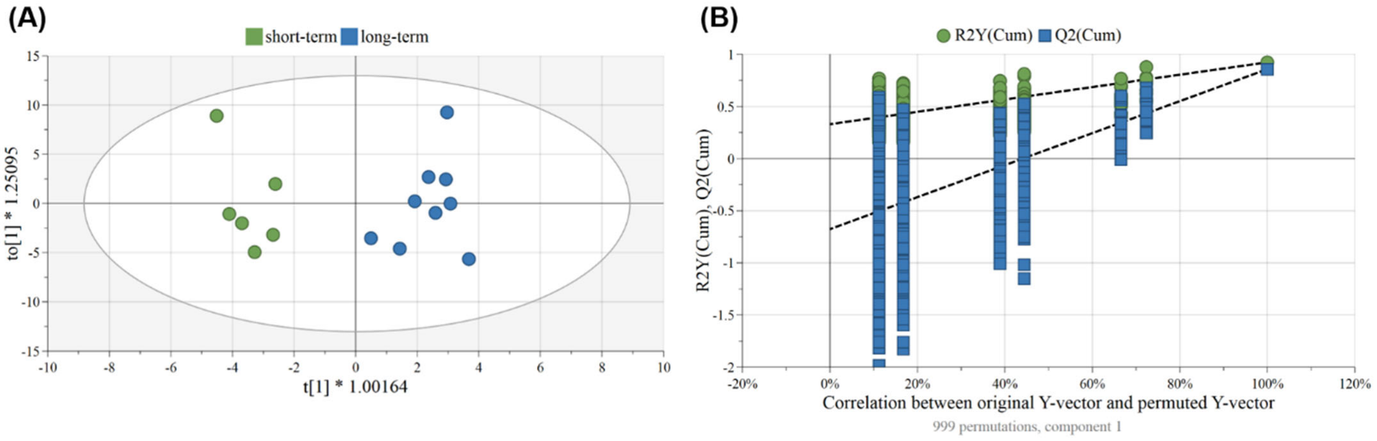

Table S1. List of identified metabolites.

No.	Metabolite	KEGG ID	Retention index	Retention time (min)	Target m/z	Fragmentation ion (m/z)	TMS[1]	CV (%)	Identification	
									library	STD[4]
1	Lactic acid	C00186	1071	9.16	73	73, 147, 117, 45	2	18.2	O	
2	Alanine	C00041	1106	10.71	116	116, 73, 147, 117	2	23.9	O	O
3	Valine	C00183	1216	15.82	144	144, 73, 147, 218	2	26.8	O	O
4	Urea	C00086	1251	17.7	73	147, 73, 189, 171	2	18.8	O	
5	2-Aminoethanol	C00189	1266	18.51	174	174, 73, 100, 147	3	13.5	O	
6	Leucine	C00123	1274	18.99	158	158, 73, 102, 147	2	25.4	O	O
7	Glycerol	C00116	1279	19.21	73	73, 147, 205, 117, 103	3	15.5	O	
8	Isoleucine	C00407	1293	20	158	158, 73, 218, 147	2	17.5	O	O
9	Glycine	C00037	1303	20.51	174	174, 73, 147, 86, 248	3	15.9	O	
10	Succinic acid	C00042	1316	21.15	147	147, 73, 75, 247	2	16.2	O	
11	Fumaric acid	C00122	1354	23.03	245	73, 147, 245, 75	2	16	O	
12	Serine	C00065	1363	23.51	73	73, 204, 218, 147, 100	3	11.4	O	O
13	Threonine	C00188	1387	24.7	73	73, 117, 218, 219, 101	3	8.2	O	
14	beta alanine	C00099	1426	26.49	248	73, 147, 174, 248, 45	3	15.7	O	
15	Aminomalonic acid	C00872	1470	28.44	73	73, 147, 218, 45, 86	3	18.1	O	
16	Malic acid	C00149	1492	29.44	73	73, 147, 233, 45	3	10.7	O	
17	Pyroglutamic acid	C01879	1514	30.38	156	73, 156, 147, 45, 230, 258	2	10.6	O	
18	Aspartic acid	C00049	1523	30.74	73	73, 232, 100, 147	3	19.3	O	
19	Creatinine	C00791	1532	31.69	115	115, 73, 143, 100, 329	3	8.7	O	O
20	Cysteine	C00097	1554	32.02	73	73, 220, 218, 100	3	17.7	O	O
21	Phenylalanine	C00079	1617	34.65	73	73, 218, 192, 147, 100	2	18.4	O	
22	Glutamic acid	C00025	1621	34.83	73	73, 246, 128, 147	3	19.1	O	O
23	Ribose	C00121	1681	37.36	73	73, 103, 217, 307	4	14.9	O	O
24	alpha-Glycerophosphate	C00093	1767	40.75	73	73, 299, 357, 147	4	12.5	O	
25	Glutamine	C00064	1774	41.01	73	73, 156, 155, 147, 245	3	14.1	O	
26	3-Phosphoglyceric acid	C00197	1810	42.31	73	73, 299, 147, 227, 101, 357	4	20.1	O	
27	Citric acid	C00158	1821	42.69	73	73, 273, 147, 75, 45	4	15.2	O	O
28	d-Erythrotetrofuranose		1830	42.96	73	73, 147, 218, 191	3	14.6	O	
29	Fructose[2)]	C00095	1874	44.41	103	73, 103, 217, 147, 307	5[3)]	15.9	O	O
30	Fructose[2)]	C00095	1884	44.72	103	73, 103, 217, 147, 307	5[3)]	18.2	O	O
31	Mannose	C00159	1888	44.83	73	73, 147, 129, 103, 319	5[3)]	20.1	O	O
32	Glucose[2)]	C00031	1897	45.15	73	73, 147, 319, 205, 103	5[3)]	16.5	O	O
33	Glucose[2)]	C00031	1914	45.64	73	73, 147, 319, 205, 103	5[3)]	18.3	O	O
34	Pantothenic acid	C00864	1987	47.77	73	73, 103, 117, 157, 291	3	17.8	O	
35	Myo-Inositiol	C00137	2086	50.66	73	73, 147, 217, 305, 191	6	17.8	O	
36	Fructose 1-phosphate	C01094	2295	54.69	73	73, 387, 103, 147, 299	6[3)]	29.2		O
37	myo-Inositiol 1-phosphate	C04006	2407	56.24	73	73, 318, 315, 147, 299	7	23.4	O	
38	Inosine	C00294	2567	58.15	73	73, 217, 230, 245, 103	4	18.6	O	
39	Sucrose	C00089	2632	58.84	73	73, 361, 147, 217, 103	8	21.4	O	

1) Number of trimethylsilyation in each compound
2) Peak splitted
3) Methoxiaminated
4) Confirmed by standard compound

Table S2. Statistical results of comparison of control and HBB exposure group.

Metabolites	VIP (> 1)	Metabolites	*p*-value (< 0.05)	Metabolites	Fold change (> 1.2 or < 0.8)
myo-Inositol 1-phosphate	2.06	Urea	2.6×10^{-4}	Urea	0.679
Urea	1.69	myo-Inositol 1-phosphate	5.2×10^{-4}	myo-Inositol 1-phosphate	0.684
Glutamine	1.58	Glucose	4.7×10^{-3}	Mannose	0.711
Glucose	1.41	beta alanine	0.041	Glucose	0.721
d-Erythrotetrofuranose	1.32	Inosine	0.045	3-Phosphoglyceric acid	0.728
Fructose 1-phosphate	1.30			Inosine	0.753
Cysteine	1.19			Fructose	0.788
Phenylalanine	1.09			Sucrose	1.383
Creatinine	1.06				
Fumaric acid	1.05				
Succinic acid	1.03				

Table S3. Metabolite pathway enrichment analysis affected by short-term and long-term exposure comparisons.

Pathway Name	Match Status	*p*-value	FDR	Impact
Valine, leucine and isoleucine biosynthesis	3/8	4.905E-5	0.0020601	0.0
Arginine biosynthesis	3/14	3.0695E-4	0.0085946	0.11675
Pantothenate and CoA biosynthesis	3/19	7.9167E-4	0.015548	0.0
Fructose and mannose metabolism	3/20	9.255E-4	0.015548	0.12802
Alanine, aspartate and glutamate metabolism	3/28	0.0025282	0.035395	0.42068
Histidine metabolism	2/16	0.011023	0.11574	0.0
Galactose metabolism	2/27	0.030179	0.26276	0.0
Glutathione metabolism	2/28	0.032306	0.26276	0.02309
Phenylalanine, tyrosine and tryptophan biosynthesis	1/4	0.040694	0.26276	0.5
Glyoxylate and dicarboxylate metabolism	2/32	0.041386	0.26276	0.0
Glycine, serine and threonine metabolism	2/33	0.043793	0.26276	0.0029
Cysteine and methionine metabolism	2/33	0.043793	0.26276	0.09592

Table S4. 10 individual GA runs clustering information table, E = binding energy (kcal/mol); Ki = inhibition constant (μM)

	GPT		PAH		RK		CAH		BCAT	
	E	KI	E	KI	E	KI	E	KI	E	KI
1	-5.14	169.38	**-6.92**	**8.41**	-5.64	73.77	-5.07	193.77	-4.96	231.78
2	-5.02	210.83	-5.38	114.56	-5.29	131.59	-5.50	-	-4.99	221.34
3	**-5.76**	**59.51**	-5.91	46.90	-5.12	176.46	-4.92	245.85	-5.44	102.85
4	-4.77	316.80	-5.04	201.68	-5.58	81.12	-5.10	184.18	-4.62	408.38
5	-5.17	161.50	-5.51	-	-4.88	264.52	-5.39	111.49	-4.72	349.02
6	-4.81	300.53	-4.77	317.41	-4.58	440.67	-5.00	215.61	-4.94	240.83
7	-4.84	283.35	-5.77	58.83	-4.76	-	-5.56	83.75	-5.10	182.54
8	-4.86	272.30	-5.67	69.48	-5.51	91.71	**-5.59**	**79.65**	-4.70	-
9	-5.00	214.69	-6.54	16.09	**-6.52**	**16.73**	-5.28	135.26	-4.49	512.49
10	-5.12	176.38	-4.56	454.38	-5.75	61.05	-5.60	79.12	**-6.30**	**23.95**
